# Flare levels after intravitreal injection of brolucizumab for diabetic macular edema

**DOI:** 10.1007/s00417-024-06374-4

**Published:** 2024-01-13

**Authors:** Yushi Ichihashi, Yoshihiro Takamura, Takao Hirano, Masahiko Shimura, Keisuke Yoneda, Keiichiro Konno, Yutaka Yamada, Masakazu Morioka, Makoto Gozawa, Takehiro Matsumura, Masaru Inatani

**Affiliations:** 1 Department of Ophthalmology, Faculty of Medical Sciences, University of Fukui, Eiheiji-Cho, Yoshida-Gun, Fukui-Ken, 910-1193 Japan; 2 Department of Ophthalmology, Shinshu University School of Medicine, Matsumoto, Japan; 3 Department of Ophthalmology, Tokyo Medical University Hachioji Medical Center, Tokyo, Japan; 4 Department of Ophthalmology, National Defense Medical College, Tokorozawa, Japan; 5 Department of Ophthalmology, Mie University Graduate School of Medicine, Tsu, Japan

**Keywords:** Diabetic macular edema, Anterior flare intensity, Brolucizumab, Inflammation, VEGF

## Abstract

**Purpose:**

This study aimed to evaluate anterior flare intensity (AFI) after intravitreal injection of brolucizumab (IVBr) in patients with diabetic macular edema (DME), and to identify the factors associated with the change of AFI after IVBr.

**Methods:**

This prospective multicenter study was conducted at five sites in Japan for patients with DME who underwent a single IVBr. AFI and central retinal thickness (CRT) were measured using a laser flare meter and spectral-domain optical coherence tomography, respectively, at weeks 0 and 6.

**Results:**

Sixty-five patients (phakia, 37 eyes; pseudophakia, 28 eyes) were enrolled. Six weeks after IVBr, CRT and best-corrected visual acuity significantly improved (*p* < 0.0001). AFI (*p* = 0.0003) and age (*p* = 0.0054) were significantly higher in patients with pseudophakic eyes than those with phakic eyes. The AFI of the phakic eyes decreased after IVBr (*p* = 0.043). As the AFI before injection is higher (*p* = 0.0363) and the age is lower (*p* = 0.0016), the AFI decreases after IVBr. There was a significant positive correlation between the rates of change in CRT and AFI (*p* = 0.024).

**Conclusion:**

After IVBr, AFI decreases in phakic eyes but not in pseudophakic eyes. The age, AFI and CRT before injection and changes of CRT are involved in the change in AFI after IVBr.


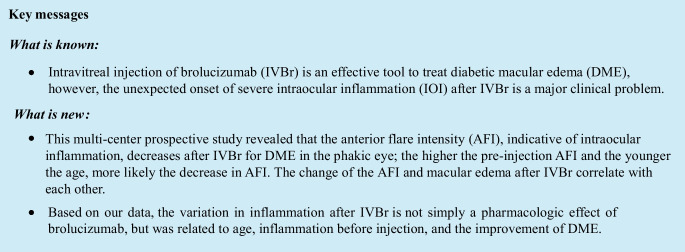


## Introduction

Diabetic macular edema (DME) causes visual impairment and distorted vision in patients with diabetic retinopathy (DR) due to increased permeability of the perifoveal capillaries or microaneurysms and disruption of the blood–retinal barrier (BRB) [1]. The etiology of DME is complex, and chronic inflammation is involved in its response to hypoxic conditions [2–4]. The levels of angiogenic mediators, such as vascular endothelial growth factor (VEGF), and inflammatory cytokines, such as interleukin (IL)-6, IL-8, tumor necrosis factor-α, and intercellular adhesion molecule-1, are elevated with the onset and progression of DME in both vitreous and aqueous humors of patients with DME [4, 5].

Currently, intravitreal injection of anti-VEGF agents is the gold standard treatment for DME [2, 6]. In Japan, four types of anti-VEGF agents, including ranibizumab, aflibercept, faricimab, and brolucizumab, are approved for use. Brolucizumab (Beovu®, Novartis, Basel, Switzerland) was launched in June 2022 for DME. It is a humanized monoclonal single-chain variable fragment that is the smallest functional unit of an antibody that binds to and inhibits VEGF-A. Brolucizumab has a higher binding affinity to VEGF-A isoforms than bevacizumab or ranibizumab [7, 8]. The KESTREL and KITE studies demonstrated that intravitreal injection of brolucizumab (IVBr) was effective in the robust gain of visual acuity and anatomical improvement [9]. Moreover, the intervals of brolucizumab injection are prolonged up to 16 weeks; thus, the treatment burden on patients may be reduced [10].

Although IVBr is expected to have favorable outcomes in DME treatment, several case reports have shown the onset of intraocular inflammation (IOI), including retinal vasculitis and vessel occlusion, after IVBr for neovascular age-related macular degeneration (nAMD) and DME [11–14]. Sub-Tenon’s capsule triamcinolone acetonide may be effective to treat and prevent brolucizumab-associated inflammation [15, 16]. Careful monitoring of anterior inflammation and IOI is required for early diagnosis of brolucizumab-related ocular complication and treatment. DME is characterized by intraretinal and subretinal accumulation of fluid due to the destruction of the BRB and is frequently accompanied by dysfunction of the blood–aqueous barrier (BAB), which correlates with anterior flare intensity (AFI). A laser flare meter is a useful tool for measuring the AFI and indicating anterior inflammation [17].

The analysis of AFI measurements after IVBr is important in clinical practice to understand the inflammatory reactions induced by brolucizumab in DME. Inflammatory response after IVBr may be influenced by several factors such as age, lens status, and the degree of DME and DR. Investigation of these associations may provide us a significant information in the interpretation of AFI change after IVBr. In this prospective multicenter study, we aimed to evaluate the alterations in AFI after a single IVBr in the eyes of patients with DME and investigate the factors influencing AFI after IVBr.

## Methods

This prospective multicenter study was conducted in accordance with the tenets of the Declaration of Helsinki. We collected data from five clinical centers in Japan. This study was approved by the Institutional Review Boards (IRBs) of the University of Fukui, University of Shinshu, National Defense Medical College, Hachioji Medical Center, and University of Mie (IRB number: 2022150, date of approval: December 16, 2022). The protocol, safety, and efficacy implications of the interventions were explained, and all patients provided written informed consent prior to enrollment. This study was registered in the University Hospital Medical Information Network Clinical Trials Registry (UMIN-CTR) of Japan (ID: UMIN000050071, date of access and registration: February 1, 2023).

Patients with type 2 diabetes and central macular thickening, defined as central retinal thickness (CRT) ≥ 300 μm in the central subfield based on spectral-domain optical coherence tomography (SD-OCT) with DME, were included in this clinical study. Leakage from retinal capillaries and microaneurysms corresponding to macular edema were identified using fluorescein angiography. In both eyes, the first treated eye was used as data. The main exclusion criteria were as follows: (1) age < 20 years; (2) focal/grid photocoagulation or pan-retinal photocoagulation (PRP) within the past 6 months; (3) active IOI or infection in either eye; (4) uncontrolled glaucoma in either eye; (5) enrollment history of intravitreal injections of angiogenesis inhibitors or steroids within 3 months of registration; (6) history of stroke; (7) systolic blood pressure > 160 mmHg, diastolic blood pressure > 100 mmHg, or untreated hypertension; and (8) glycosylated hemoglobin level ≥ 10%.

All patients underwent a slit-lamp examination, dilated fundus examination, fundus photography, best-corrected visual acuity (BCVA) measurement (Snellen), intraocular pressure (IOP) measurement, OCT, and flare photometry. BCVA measured using a Landolt chart was converted to the logarithm of the minimum angle of resolution.

AFI was measured using a laser flare meter (FM-600; Kowa Co. Ltd., Tokyo, Japan) and reported as photons per millisecond. Measurements were performed before treatment (baseline) and 6 weeks after injection. For AFI, measurements were performed in a darkened room at least 20 min after the dilation of the pupil with 0.5% tropicamide and 0.5% phenylephrine hydrochloride (Mydrin P; Santen, Tokyo, Japan), repeated 10 times, and subsequently averaged to obtain the final value. CRT was measured at the same time points using SD-OCT (Cirrus OCT; Carl Zeiss Meditec, Dublin, CA, USA). To minimize measurement errors, all the tests were performed by an experienced examiner (Y.Y.). BCVA, CRT, and AFI were measured before and 6 weeks after a single IVBr. The rate of change in CRT or AFI was calculated by dividing the difference between the pre- and post-injection values by the pre-injection value and expressing it as a percentage.

Intravitreal injections were performed in a standard manner by a trained ophthalmologist (Y.I.) using 0.4% oxybuprocaine hydrochloride (0.4% benoxyl ophthalmic solution; Santen Co. Ltd., Osaka, Japan) and 2% xylocaine as anesthetics and povidone iodine for sterilization. After stabilizing the eyelids with an eyelid speculum, the anti-VEGF agent brolucizumab (Beovu® Novartis Pharma, Basel, Switzerland; 6 mg in 0.05 mL) was injected into the vitreous cavity using a 30-gauge needle. Antibiotic eye drops were not used before or after the injection.

We divided the patients into three groups according to the International Classification of Diabetic Retinopathy severity: mild-moderate non-proliferative diabetic retinopathy (NPDR), severe-NPDR, and PDR [18]. Multiple regression analysis was performed to investigate the relationship between the change of the AFI at 6 weeks after IVBr and other factors including age, the status of lens (scored 0: phakia, 1: pseudophakia), the value of AFI and CRT at baseline, and the degree of DR (scored 1: mild and moderate NPDR, 2: severe NPDR, 3: PDR). The patients were also divided into phakic and pseudophakic groups based on their history of cataract surgery.

A sample size of 55 participants, while accounting for an approximately 10% dropout rate, would have provided 80% power, at a 1-sided α-level of 0.025, as calculated by G*power. Statistical analyses were performed using JMP (SAS Institute Inc., Tokyo, Japan). Data were presented as means ± standard deviations of the means. The Mann–Whitney U test was used to compare continuous variables between phakic and pseudophakic eyes. Significant differences between the different time points were analyzed using the Wilcoxon signed-rank test. Differences were considered statistically significant at p < 0.05.

## Results

**Fig. 1:**
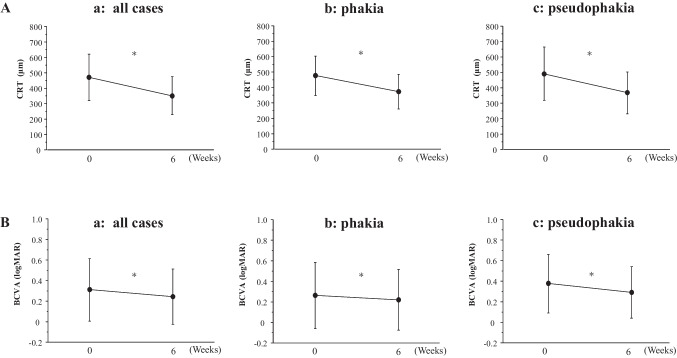
Change in (**A**) central retinal thickness (CRT) and (**B**) best-corrected visual acuity after intravitreal injection of brolucizumab in all patients (**a**), patients with phakic eyes (**b**), and patients with pseudophakic eyes (**c**). The data are shown as means ± standard deviations. ^*^p < 0.05 (CRT compared with baseline)

In total, 65 patients (phakia, 37 eyes; pseudophakia, 28 eyes) were included in this study. None of the patients experienced adverse events after the injection, including retinal detachment, endophthalmitis, vitreous hemorrhage, or IOI. The patient demographic data are presented in Table 1. Changes in CRT and BCVA after IVBr are presented in Fig. 1A and B, respectively (a, all patients; b, phakia; c, pseudophakia). A significant decrease in CRT was observed at 6 weeks in all patients (*p* < 0.0001), patients with phakic eyes (*p* < 0.0001), and patients with pseudophakic eyes (*p* = 0.0003). BCVA significantly improved in all patients (*p* < 0.0001), patients with phakic eyes (*p* = 0.0003), and patients with pseudophakic eyes (*p* = 0.0057). No significant difference in IOP was observed before and 6 weeks after the injection in either the phakic or pseudophakic eyes.

**Table 1:** Baseline characteristics at the time of registration

Age (years)	62.2 ± 12.4
Gender (male/female)	49 / 16
Duration of diabetes mellitus (years)	10.5 ± 1.2
Hemoglobin A1c (%)	7.6 ± 0.9
Insulin therapy	28 (43.1%)
Serum creatinine	1.92 ± 3.74
Lens status (phakia/pseudophakia)	37 / 28
Severity of diabetic retinopathy	
Mild NPDR	2
Moderate NPDR	18
Severe NPDR	30
PDR	15

*NPDR* non-proliferative diabetic retinopathy, *PDR* proliferative diabetic retinopathy

**Fig. 2:**
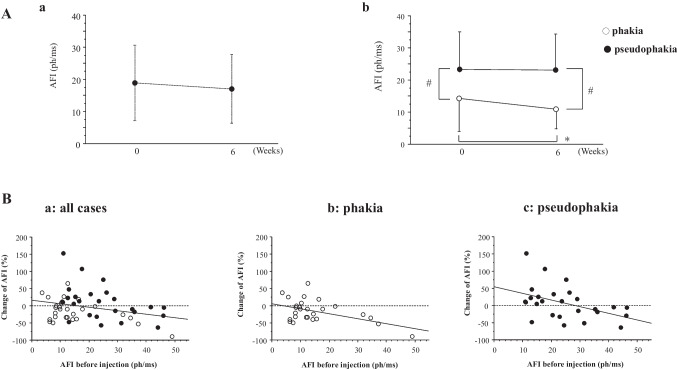
**A** Change in anterior flare intensity (AFI) after intravitreal injection of brolucizumab in all patients (**a**) and in the patients with phakic and pseudophakic eyes (**b**). The data are shown as means ± standard deviations. ^*^p < 0.05 (AFI compared with baseline). ^#^*p* < 0.05 (phakia group vs. pseudophakia group). **B** Linear correlation between the change of AFI and the AFI before injection. Significant correlations were observed in (**a**) all patients, (**b**) patients with phakic eyes, and (**c**) patients with pseudophakic eyes

The value of AFI before injection was 19.3 ± 12.2 ph/ms. After 6 weeks, the AFI did not significantly change (*p* = 0.094) in all patient group (Fig. 2Aa). The AFI in the pseudophakic eyes was significantly higher than that in the phakic eyes at baseline (*p* = 0.0003) and at 6 weeks (*p* = 0.0001) (Fig. 2Ab). The AFI significantly decreased in the phakic eyes (*p* = 0.043) but not in the pseudophakic eyes (p = 0.81). An increase on AFI after brolucizumab injection were seen in 57.1% of the pseudophakic eye group and only 16.2% in the phakic eye group. There was a significant negative correlation between the AFI at baseline and its rate of change in all patients (*p* = 0.0363, Y = 16.371 – 1.019 X, R^2^ = 0.08), patients with phakic eyes (*p* = 0.0098, Y = 5.317 – 1.432X, R^2^ = 0.24), and patients with pseudophakic eyes (*p* = 0.022, Y = 55.134 – 1.933X. R^2^ = 0.21) (Fig. 2B). This indicates that the higher the pre-injection AFI, the greater the decrease in the post-injection flare value.

We carried out multiple regression analysis to explore the relating factors with the change of the AFI at 6 weeks after IVBr. As shown in Table 2, the age (*p* = 0.0052), the status of lens (*p* = 0.016), the value of AFI (*p* = 0.0002), and CRT (*p* = 0.043) at baseline were significantly correlated with the change rate of AFI at 6 weeks after IVBr.

**Table 2:** Multiple regression analysis relating change of AFI

Factor	Standardized regression efficient	p-value
Age	0.357	0.0052
Lens status	0.329	0.0159
AFI before injection	-0.527	0.0002
CRT before injection	0.244	0.0433
Grade of diabetic retinopathy	-0.067	0.5731

*AFI* anterior flare intensity, *CRT* central retinal thickness

**Fig. 3:**
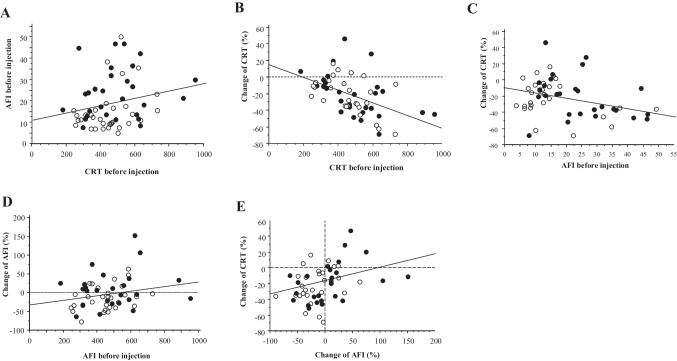
Linear correlation between anterior flare intensity (AFI) and central retinal thickness (CRT). White dot; phakia, black dot; pseudophakia. **A** Linear correlation between AFI and CRT before injection. **B** Linear correlation between CRT at baseline and the change ratio of CRT after injection. **C** Linear correlation between the change ratio of CRT after injection and AFI before injection. **D** Linear correlation between the change ratio of AFI after injection and AFI before injection. **E** Linear correlation between the change of CRT and AFI

We examined the relationship between baseline CRT and AFI (Fig. 3A), and the significant correlation was found (*p* = 0.042, Y = 9.558 + 0.019X, R^2^ = 0.061). The change in CRT also significantly correlated with the CRT at baseline (*p* = 0.0001, Y = 13.725—0.076X, R^2^ = 0.231) (Fig. 3B), and the AFI at baseline in all patients (*p* = 0.021, Y = –10.332 – 0.631X, R^2^ = 0.092) (Fig. 3C). The change of the AFI after injection was also significantly correlated to the CRT at baseline (*p* = 0.042, Y = -32.307 + 0.06X; R^2^ = 0.075) (Fig. 3D). Next, we investigated the relationship between the change of CRT and AFI (Fig. 3E), and a significant correlation was found (*p* = 0.024, Y = –21.482 + 0.17 X, R^2^ = 0.092). The percentage of patients with CRT reduction of more than 20% after injection was 51.4% (19/37) in phakic eyes and 46.4% (13/28) in pseudophakic eyes.

**Fig. 4:**
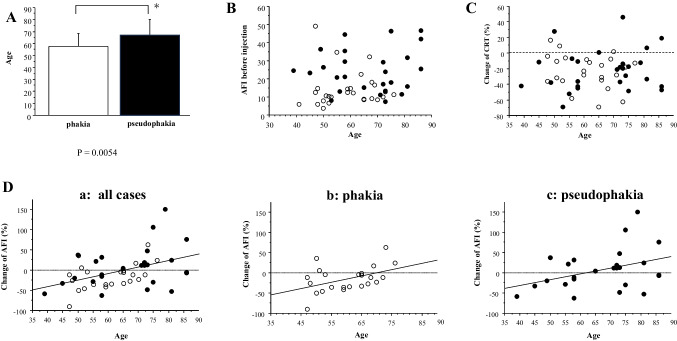
**A** Comparison of age between patients with phakic and pseudophakic eyes. ^*^p < 0.05 (phakia group vs. pseudophakia group). Linear correlation between the age and anterior flare intensity (AFI) before injection (**B**) or change of central retinal thickness (**C**). No significant correlation was found. (**D**) Linear correlation between the change of AFI and the AFI before injection. Significant correlations were observed in (**a**) all patients, (**b**) patients with phakic eyes, and (**c**) patients with pseudophakic eyes. White dot, phakia; black dot, pseudophakia

The patients with pseudophakic eyes were older than those with phakic eyes (*p* = 0.0054) (Fig. 4A). We aimed to determine whether age played a role in the differences in AFI values between phakic and pseudophakic eyes. Age was not significantly correlated with the AFI before injection (p = 0.14) (Fig. 4B) or the change in CRT (*p* = 0.79) (Fig. 4C). There was a significant correlation between age and changes in AFI in patients with phakic eyes (*p* = 0.0301, Y = –109.805 + 1.578 X, R^2^ = 0.205), patients with pseudophakic eyes (*p* = 0.041, Y = –87.075 + 1.411X, R^2^ = 0.144), and all patients (*p* = 0.0016, Y = –104.449 + 1.601X, R^2^ = 0.19) (Fig. 4D). The ages of the patients whose AFI change increased or decreased by more than 20% after injection were 68.3 ± 12.4 and 58.2 ± 11.3 years, respectively, and the age difference was significant (*p* = 0.033).

**Fig. 5:**
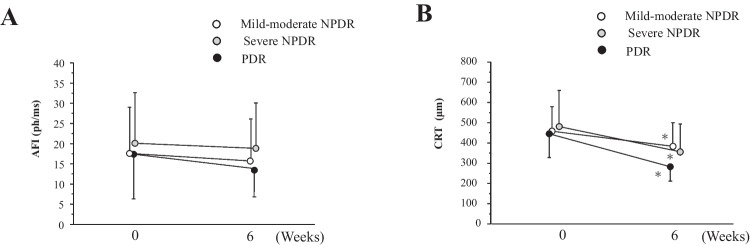
Change in (**A**) anterior flare intensity (AFI) and (**B**) central retinal thickness (CRT) after intravitreal injection of brolucizumab in the patients with mild-moderate NPDR (white circle), severe NPDR (gray circle), and PDR (black circle). The data are shown as means ± standard deviations. ^*^p < 0.05 (CRT compared with baseline)

In this case series, the numbers of mild and moderate NPDR, severe NPDR, and PDR were 20 (mild 2, moderate 18), 30 and 15, respectively. PRP had already been performed in 20% (6/30) of severe NPDR and in all PDR cases. There were no significant differences in age, the rates of pseudophakic eye, AFI or CRT before injection among the groups. We found no significant difference of the AFI before and 6 weeks after IVBr in each DR group (Fig. 5A). On the other hand, the CRT significantly decreased in mild-moderate NPDR (*p* = 0.0019), severe NPDR (*p* = 0.0006), and PDR (*p* = 0.0022) group (Fig. 5B).

## Discussion

In this study, we analyzed the changes in AFI, which indicates inflammation, after a single IVBr. As previously reported [19], the AFI was significantly higher in pseudophakic eyes than in phakic eyes before injection. Cataract surgery alters the aqueous humor microenvironment by disrupting the BAB and upregulating inflammatory cytokines, such as IL-8 and monocyte chemotactic protein 1 [20, 21]. A significant decrease in AFI was observed in phakic eyes but not in pseudophakic eyes. The higher the AFI value at baseline, the more the flare decreased after injection in both phakic and pseudophakic eyes. In the pseudophakic eyes, the decrease in AFI values was not significant despite higher AFI values before injection. This may be because the percentage of the patients whose AFI values increased after injection was 57.1% in the pseudophakic eye group and only 16.2% in the phakic eye group, indicating a greater case-to-case variability in AFI values in the pseudophakic eyes. In DME, the pharmacological effect of brolucizumab does not simply reduce AFI after injection. Some factors appear to increase AFI, especially in pseudophakic eyes. The reason why AFI is elevated in the pseudophakic eye is unclear. The amount and the kind of cytokines present in the pseudophakic eye may differ from those in the phakic eye, resulting in a differential inflammatory response. It is also possible that the reaction to the invasion of the injection itself, even minimally, may be different between phakic and pseudophakic eyes.

The results of multiple and single regression analysis showed not only the lens status but also the age, the AFI and CRT before injection were associated with the change of AFI, independently. In both phakic and pseudophakic eyes, the older the patient, the higher was the AFI value after injection. Patients who underwent cataract surgery were older than those with lens eyes, which may explain why the flare values tended to increase after injection in pseudophakic eyes. Our data demonstrated that age was not correlated with flare values before injection in patients with DME. Therefore, the effect of age on AFI is likely to be limited to post-injection changes and not to the pre-injection status. An age-related alteration in the immune response may be involved in the increased AFI levels caused by brolucizumab injection. To compare AFI changes in pseudophakic and phakic eyes while excluding the effect of age, both age-matched groups should be compared in the same number of cases and further studies with larger numbers of patients are needed.

In this study group of patients with DME, AFI was 19.3 ± 12.2 ph/ms before injection, which was higher than normal (< 10 ph/ms). At baseline, the AFI was correlated with the value of the CRT indicating the high AFI before injection was probably reflected with the inflammation due to DME. Our data also showed that the changes in CRT and AFI after injection also correlated with each other. Taken together, the improvement of edema after IVBr may be accompanied with inhibition of anterior inflammation, which is reflected as a decrease in AFI. It is suggested that the vascular permeability was decreased by brolucizumab and the DME improved, the inflammation indicated by AFI also decrease. Conversely, when edema worsens despite brolucizumab injections, it may be accompanied by advanced disruption of BAB. The cytokine variability may differ between cases in which both edema and inflammation improve and those in which they worsen after injection. Further analysis, including measurement of inflammatory cytokines before and after injection, will be necessary to clarify this issue.

AFI values have been reported to increase in correlation with the severity of DR [22]. However, in our analysis, there was no significant difference in AFI between NPDR and PDR at baseline. In our study, all PDR patients were treated with PRP, and the proliferative activity may have inhibited. Regardless of DR severity, AFI did not change significantly after IVBr, while the retinal thickness improved. DR severity does not appear to be a significant factor in the change in AFI after IVBr.

For ranibizumab, another anti-VEGF drug, Imazeki et al. reported a significant decrease in AFI 1 month after ranibizumab injection; the greater the decrease, the better the visual acuity and CRT [22]. Shiraya et al. also reported a significant decrease in flare values 2 weeks after ranibizumab administration [23]. For aflibercept, they also reported a transient increase on the day after injection and no change at 2 weeks post-injection compared with pre-injection [22, 23]. There was no significant relationship between AFI values and percent change in CRT for any of the drugs. In our previous report, similar to Shiraya et al., there was a transient increase in AFI 1 day after aflibercept injection and no significant change in flare values before or after injection at 1 week or 1 month after either ranibizumab or aflibercept injection [24, 25]. The reason for the dissociation of these data may be that the phakic and pseudophakic eyes were not analyzed separately in each report, and the periods between injection and measurement were not equal. In particular, the injection interval for ranibizumab and aflibercept was 4 weeks, whereas that for brolucizumab was 6 weeks, making a simple comparison with the present study difficult.

In the treatment of nAMD, rare cases of IOI after brolucizumab injections were reported in 2020 [11, 12]. Immunopathological theories have been proposed as a possible mechanism for this complication [26]. In brolucizumab-naïve patients, anti-brolucizumab antidrug antibody responses were detected before treatment, suggesting prior exposure of the immune system to structurally similar proteins. Patients with IOI show a meaningful T-cell response upon reinjection of brolucizumab. Possibly due to the time required for the immune response, IOI occurs more frequently in the first few months after the initial brolucizumab injection [11–13, 27]. In this study, no patients showed IOI after IVBr, and thus there is no data about the variation of AFI in patients who develop IOI. An analysis of the variability of AFI in patients who develop IOI after injection is needed.

## Data Availability

The datasets generated during and/or analyzed during the current study are available from the corresponding author on reasonable request.
